# Pupillometric Complexity and Symmetricity Follow Inverted-U Curves Against Baseline Diameter Due to Crossed Locus Coeruleus Projections to the Edinger-Westphal Nucleus

**DOI:** 10.3389/fphys.2021.614479

**Published:** 2021-02-11

**Authors:** Sou Nobukawa, Aya Shirama, Tetsuya Takahashi, Toshinobu Takeda, Haruhisa Ohta, Mitsuru Kikuchi, Akira Iwanami, Nobumasa Kato, Shigenobu Toda

**Affiliations:** ^1^Department of Computer Science, Chiba Institute of Technology, Chiba, Japan; ^2^National Center of Neurology and Psychiatry, Department of Preventive Intervention for Psychiatric Disorders, National Institute of Mental Health, Tokyo, Japan; ^3^Research Center for Child Mental Development, Kanazawa University, Ishikawa, Japan; ^4^Department of Neuropsychiatry, University of Fukui, Fukui, Japan; ^5^Uozu Shinkei Sanatorium, Uozu, Japan; ^6^Faculty of Letters, Ryukoku University, Kyoto, Japan; ^7^Medical Institute of Developmental Disabilities Research, Showa University, Tokyo, Japan; ^8^Department of Psychiatry & Behavioral Science, Kanazawa University, Ishikawa, Japan; ^9^Department of Psychiatry, School of Medicine, Showa University, Tokyo, Japan; ^10^Department of Psychiatry, Showa University East Hospital, Showa University, Tokyo, Japan

**Keywords:** pupillometry, complexity, symmetricity, neural model, non-linear dynamics, hippus

## Abstract

In addition to photic reflex function, the temporal behavior of the pupil diameter reflects levels of arousal and attention and thus internal cognitive neural activity. Recent studies have reported that these behaviors are characterized by baseline activity, temporal complexity, and symmetricity (i.e., degree of symmetry) between the right and left pupil diameters. We hypothesized that experimental analysis to reveal relationships among these characteristics and model-based analysis focusing on the newly discovered contralateral projection from the locus coeruleus (LC) to the Edinger-Westphal nucleus (EWN) within the neural system for controlling pupil diameter could contribute to another dimension of understanding of complex pupil dynamics. In this study, we aimed to validate our hypothesis by analyzing the pupillary hippus in the healthy resting state in terms of sample entropy (SampEn), to capture complexity, and transfer entropy (TranEn), to capture symmetricity. We also constructed a neural model embedded with the new findings on neural pathways. The following results were observed: first, according to surrogate data analysis, the complexity and symmetricity of pupil diameter changes reflect a non-linear deterministic process. Second, both the complexity and the symmetricity are unimodal, peaking at intermediate pupil diameters. Third, according to simulation results, the neural network that controls pupil diameter has an inverted U-shaped profile of complexity and symmetricity vs. baseline LC activity; this tendency is enhanced by the contralateral synaptic projections from the LCs to the EWNs. Thus, we characterized the typical relationships between the baseline activity and the complexity and symmetricity of the pupillometric data in terms of SampEn and TranEn. Our evaluation method and findings may facilitate the development of estimation and diagnostic tools for exploring states of the healthy brain and psychiatric disorders based on measurements of pupil diameter.

## 1. Introduction

Over decades, studies of pupil-diameter behavior (pupillometry) have revealed that it reflects internal neural activity affecting arousal and attention, in addition to simple photic reflexes (Rajkowski, 1993; Gilzenrat et al., 2003; Nassar et al., 2012; Eldar et al., 2013; Ebitz et al., 2014; Hong et al., 2014; Reimer et al., 2014) (review in Aston-Jones and Cohen, 2005; McGinley et al., 2015; Szabadi, 2018). The reason is that the locus coeruleus (LC), which is the norepinephrine (NE) source for both the sympathetic pathway to the pupil dilator muscle and the parasympathetic pathway to the pupil sphincter muscle, also modulates cognitive functions (Breen et al., 1983; McCormick and Pape, 1990; Ego-Stengel et al., 2002; Hirata et al., 2006; Moxon et al., 2007; Reimer et al., 2014; Martins and Froemke, 2015; McGinley et al., 2015; Wekselblatt and Niell, 2015). The LC adjusts the exploration-exploitation tradeoff in the brain by its activity pattern, i.e., it has a tonic firing mode corresponding to the exploration process (LC activity occurs for a long term and is continuous, with a high baseline activity of 1–5 Hz) and a phasic firing mode corresponding to the exploitation process (LC activity is sparse and bursty with a low baseline activity; review in Aston-Jones and Cohen, 2005). The baseline LC activity in these firing modes is strongly reflected in pupil diameter (Rajkowski, 1993; Gilzenrat et al., 2003; Joshi et al., 2016) (review in Aston-Jones and Cohen, 2005). Several experimental studies of pupil diameter in humans and mouse models show that it represents cognitive processes in both the healthy brain (reviewed in Aston-Jones and Cohen, 2005; van der Wel and van Steenbergen, 2018) and psychiatric disorders. In the latter, pupil diameter can reflect deficits of attention or arousal or imbalances in the exploration-exploitation tradeoff, which are observed in schizophrenia (Reddy et al., 2018; Thakkar et al., 2018), autism spectrum disorder (ASD), (Anderson and Colombo, 2009; Martineau et al., 2011; Gotham et al., 2018; Bast et al., 2019), and attention-deficit hyperactivity disorder (ADHD) (Wainstein et al., 2017).

Another approach to the study of pupil behavior is mathematical modeling. Various neural models have been constructed with the aim of reproducing pupil behavior in the resting state and under various types of visual input (Usui and Stark, 1982; Longtin and Milton, 1989; Pamplona et al., 2009; Watson and Yellott, 2012; Johansson and Balkenius, 2018). Usui and Stark first described the characteristic of temporal complexity in a modeling study of the autonomous fluctuation of pupil diameter called hippus (Usui and Stark, 1982). In their model, internal fluctuating neural activity is applied to the sympathetic pathway to the pupil dilator muscle and to the parasympathetic pathway to the pupil sphincter muscle. They showed that temporal complexity as measured by the standard deviation of pupil diameter exhibits an inverted U-shape against pupil diameter. This effect results from the non-linear relationship between pupil dilation and the applied neural activity. This profile of complexity is highly congruent with experimental findings (Usui and Stark, 1982). Johansson and Balkenius constructed a more complex model of pupil control based on a neural system comprising amygdala, LC, cerebellum, and other regions (Johansson and Balkenius, 2018). Interactions among neural activities, especially cortical activities, among far-flung brain regions could reproduce several kinds of reactions of pupil diameter to stimuli and exhibit emotional and learning effects (Johansson and Balkenius, 2018).

Furthermore, recent progress in research on pupil diameter has provided new insights into the temporal patterns of pupil diameter and its controlling neural pathways (Liu et al., 2017; Poynter, 2017; Wahn et al., 2017; Artoni et al., 2019; Nakamura et al., 2019; Piu et al., 2019). In particular, it has been discovered that the parasympathetic pathways from the LC project are inhibitory to both the ipsilateral and contralateral parts of the Edinger-Westphal nucleus (EWN) to control the pupil sphincter muscles, not only to the ipsilateral parts as previously thought (Liu et al., 2017). Nakamura et al. and Artoni et al. demonstrated that the temporal hippus patterns reflect several stages of cognitive processing (Nakamura et al., 2019) and are affected by psychiatric disorders (Artoni et al., 2019). Piu et al. showed that the hippus fluctuation reflects the non-linear dynamics of autonomous neural activity; this determinism and non-linear complexity might be utilized as a diagnostic tool for detecting the states of neural systems (Piu et al., 2019). In addition to the temporal pattern of pupil diameter, the asymmetricity between the right and left pupil diameters is informative, reflecting the attention function (Poynter, 2017; Wahn et al., 2017). In particular, Wahn et al. showed that this asymmetricity exhibits dependency on attentional load and events (Wahn et al., 2017). Poynter reported that pupil asymmetricity reflects gender, attentional load, and symptoms of ADHD such as inattention and hyperactivity (Poynter, 2017). However, the mechanisms that induce complex temporal patterns and asymmetry in pupil diameters remain unclear.

A model-based approach, which can describe internal neural activity as arising from interactions among brain regions and non-linear neural characteristics, and the approach of combining modeling with experimentation, are effective in revealing the mechanisms of complexity and asymmetricity in pupil behavior. We hypothesized that a model-based approach focusing on the newly discovered contralateral projection from the LC to the EWN combined with an experimental study of the relationship between complexity and asymmetricity would be effective in elucidating the complexity of pupil behavior. In the present study, we aimed to validate our hypothesis by analyzing the hippus in the healthy resting state and constructing a neural model embedded with the new findings on neural pathways. Concretely, we analyze the relationships between baseline pupil activity during hippus and sample entropy (SampEn) (Richman and Moorman, 2000), a measure of temporal complexity, and transfer entropy (TranEn) (Schreiber, 2000), a measure of the asymmetricity of the left and right pupil diameters. Furthermore, by constructing a neural system for controlling pupil diameter that incorporates the contralateral projections to the EWN from the LC, the influences of these projections on temporal complexity and asymmetricity were evaluated.

## 2. Materials and Methods

### 2.1. Participants

The study was performed at the Medical Institute of Developmental Disabilities Research, Showa University, Japan, and included 17 healthy participants (7 male, 10 female, average age 36.3 y, SD = 8.0, range = 22 − 51). The exclusion criteria were as follows: a current major depressive or manic-depressive episode, a history of psychosis, Wechsler full-scale intelligence quotient <80, a history of head injury with loss of consciousness, a sensory-motor handicap, or neurological illness. All participants had normal or corrected-to-normal vision and normal hearing. After providing complete explanation of the study procedures, written informed consent was obtained. All methods were performed in accordance with the Declaration of Helsinki, and the study protocol was approved by the Ethics Committee of Showa University.

### 2.2. Recording Pupil Diameters

**Figure 1:**
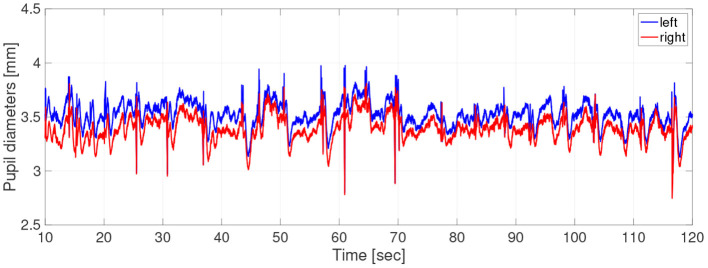
Typical example of time-series of pupil-diameters. Here, missing values due to blinks and eye movements were linearly interpolated; the low-pass filtered between 0 and 50 Hz was applied.

Participants did not use caffeine, nicotine, or any medication that might influence eye movements on the day of the experiment. For the measurements of pupil diameter, the participants sat in front of a monitor subtending 50.9 × 28.6 degrees of visual angle at a distance of 57 cm, in a lit room. The participant's head position was fixed by a chin rest. To obtain the pupil-diameter data during hippus, the participants were instructed to gaze steadily at a fixation point consisting of a black cross displayed on a screen of brightness 0.87cd/m^2^ for 2 min. The cross subtended 0.5 × 0.5 degrees of visual angle. The fixation points were generated with the Psychophysics Toolbox routines (Brainard, 1997; Pelli, 1997) for MATLAB (Version 2013b, MathWorks Ltd, http://www.mathworks.com/) and presented on a 23-inch LCD monitor (1920× 1080 pixels at 60 Hz) driven by a PC (Windows 7). During measurements, the participant's eye positions and pupil diameters were simultaneously recorded by a remote-type eye tracker (TX300; Tobii Technology, Stockholm, Sweden) with a sampling frequency of 300 Hz. In Figure 1, typical time-series of pupil diameters was shown.

In the time-series, the unmeasurable periods appeared due to the blinks and eye movements. Because the time-series in these periods disturbed the results of evaluation indexes, we chose the periods not including unmeasurable periods as much as possible by setting a short epoch length. Therefore, at the pre-processing stage, the time-series data were divided into epochs of length 2.0 s and missing values were linearly interpolated. Epochs missing more than 10% of epoch length were excluded from the analysis. Evaluations of size, complexity, and symmetricity are independently conducted at each epoch. The epochs were low-pass filtered between 0 and 50 Hz. At the end of the pre-processing stage, the epoch length is 2 s, consisting of 600 data points. The number of useable epochs per participant was average, 39.7; SD, 17.4; and range, 5–67.

### 2.3. Pupil-Diameter Analysis: Complexity

To quantify the temporal complexity of pupil diameter, we calculated the SampEn. From *N* stochastic variables {*x*_1_, *x*_2_, ⋯*x*_*N*_} normalized by z-score, *m*-dimensional vectors are constructed:

(1) $$\begin{array}{r} {\text{x}}_{i}^{m}=\left\{x_{i},x_{i+1},\cdots \;,x_{i+m-1}\right\}, \end{array}$$

The probability *C*_*m*_(*r*) is calculated over *i, j* (*i* ≠ *j*, *i, j* = 1, 2, ⋯ ) pairs of these vectors as follows:

(2) $$\begin{array}{r} C_{m}\left(r\right)=\underset{i,j\in r,i\neq j}{\sum }\frac{\left|{\text{x}}_{i}^{m}-{\text{x}}_{j}^{m}\right|}{\left(N-m+1\right)\left(N-m\right)}\;. \end{array}$$

where $\left|{\text{x}}_{i}^{m}-{\text{x}}_{j}^{m}\right|$ indicates that it is counted when the distance between any two vectors ${\text{x}}_{i}^{m}$ and ${\text{x}}_{j}^{m}$ as the norm is less than *r*. SampEn is then defined as

(3) $$\begin{array}{r} h\left(r,m\right)=-\log\frac{C_{m+1}\left(r\right)}{C_{m}\left(r\right)}. \end{array}$$

In this study, we used *r* = 0.2, *m* = 2 (Costa et al., 2002). *h*(*r, m*) increases monotonically with the complexity of the time series. In calculating the SampEn, we used PhysioToolkit at the following address: http://physionet.incor.usp.br/physiotools/sampen/ as a toolbox in MATLAB ® (Goldberger et al., 2000).

### 2.4. Pupil-Diameter Analysis: Symmetricity

To evaluate the symmetricity of the right and left pupil diameters, we used the causality between the right and left pupil diameters as captured by the TranEn. The TranEn *T*_*X*→*Y*_ from the time-series {*x*_1_, *x*_2_, ⋯ , *x*_*t*_, ⋯ } to the time-series {*y*_1_, *y*_2_, ⋯ , *y*_*t*_, ⋯ } is defined as follows (Schreiber, 2000):

(4) $$T_{X\to Y}=\sum_{y_{t+\tau },{y_{t}}^{d_{y}},{x_{t}}^{d_{x}}}p(y_{t+\tau }|{y_{t}}^{d_{y}},{x_{t}}^{d_{x}})\log\left(\frac{p(y_{t+\tau }|{y_{t}}^{d_{y}},{x_{t}}^{d_{x}})}{p(y_{t+\tau }|{y_{t}}^{d_{y}})}\right).$$

where *t* is the time index; *p*(·|·) is the conditional probability. ${x_{t}}^{d_{x}}$ and ${y_{t}}^{d_{y}}$ denote *d*_*x*_- and *d*_*y*_-dimensional delay vectors:

(5) $${x_{t}}^{d_{x}}=\left(x_{t},x_{t-\tau },\cdots ,x_{t-(d_{x}-1)\tau }\right),$$

(6) $${y_{t}}^{d_{y}}=\left(y_{t},y_{t-\tau },\cdots ,y_{t-(d_{y}-1)\tau }\right).$$

We used τ = 10 (corresponding to a period of 0.033 s), *d*_*x*_ = 5, and *d*_*y*_ = 5. If there is no causality from *X* to *Y*, *T*_*X*→*Y*_ = 0. With increasing causality from *X* to *Y*, *T*_*X*→*Y*_ becomes larger. In this study, TranEn from the right to left pupils and that from the left to right pupils were measured. High TranEn (≫0) indicates high symmetricity between the pupil diameters, while low TranEn (≈ 0) indicates the emergence of asymmetricity. In calculating the TranEn, we used HERMES as a toolbox in MATLAB® (Niso et al., 2013).

### 2.5. Neural Model of the Controller of Pupil Diameter

**Figure 2:**
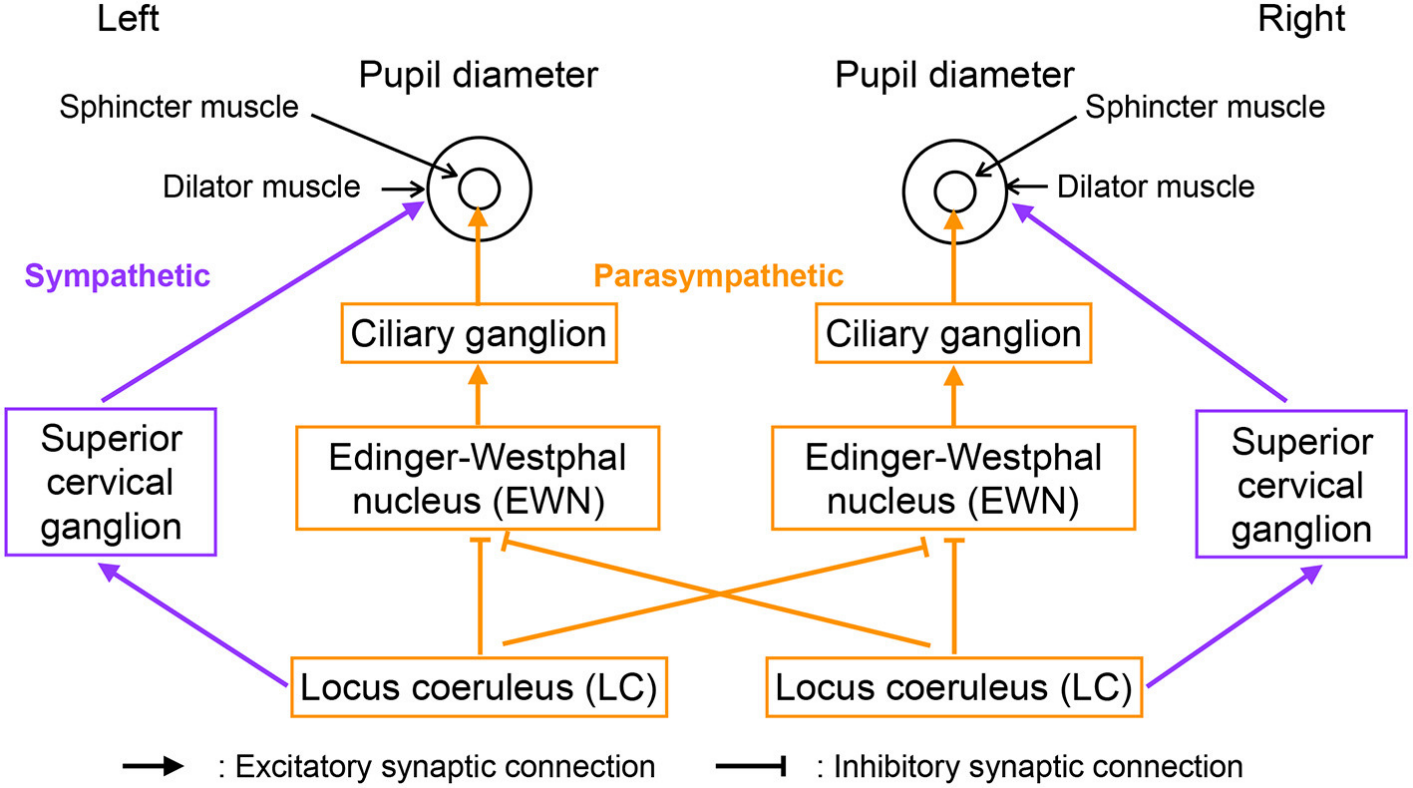
Neural pathways controlling pupil diameter.

**Figure 3:**
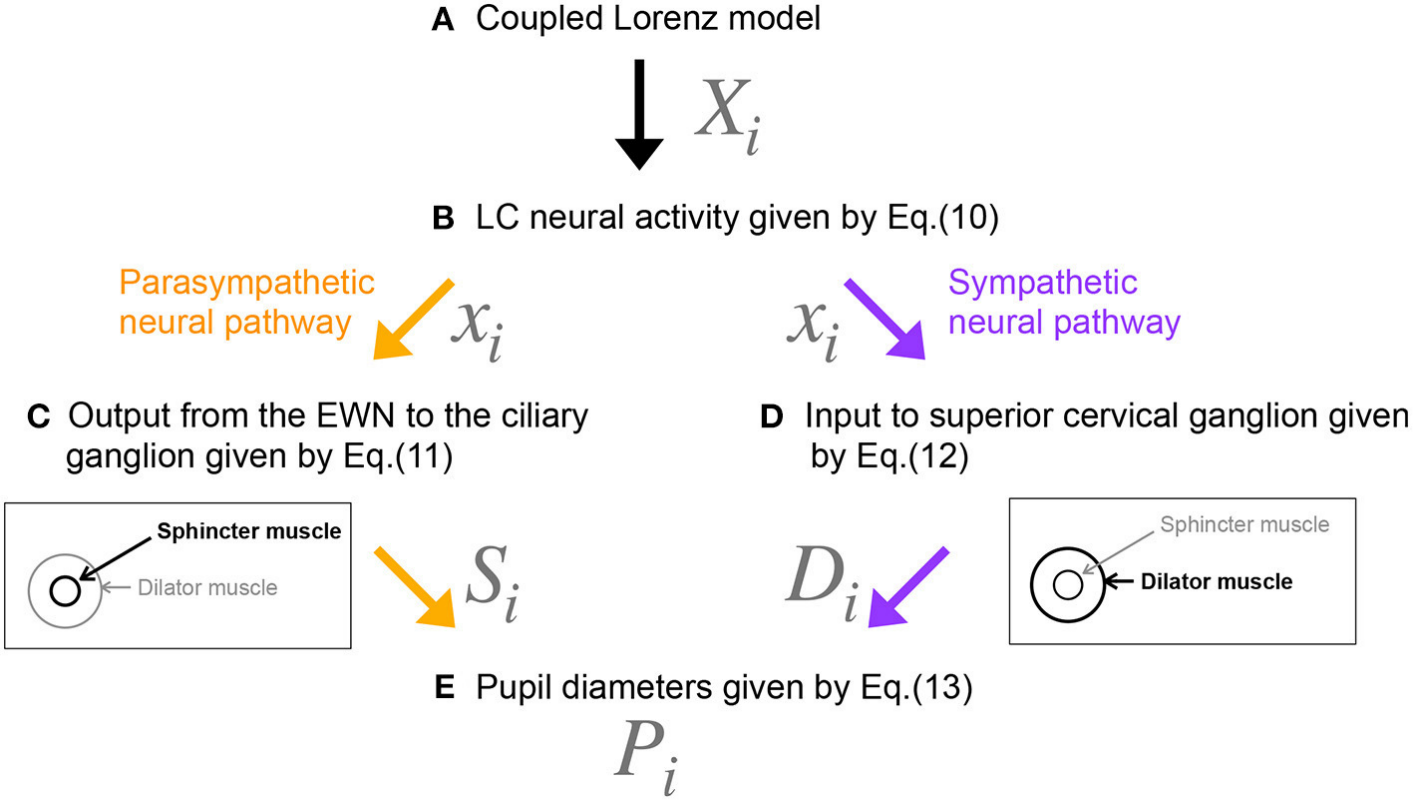
Methodological flowchart for neural model of the controller of pupil diameter. **(A)** Coupled Lorenz model. **(B)** LC neural activity given by Equation (10). **(C)** Output from the EWN to the ciliary ganglion given by Equation (11). **(D)** Input to superior cervical ganglion given by Equation (12). **(E)** Pupil diameters given by Equation (13).

Figure 2 shows our model of the neural pathways that control pupil diameter, which includes the contralateral projections from the LC to the EWN discovered by Liu et al. (2017). The LC consists of multiple synchronously firing neuron ensembles, not one globally synchronous neural population (reviewed in Totah et al., 2019). Therefore, in this study, we assumed different neural populations in the left and right LCs. Figure 3 shows an overview of the model as a methodological flowchart of the neural model of the controller of pupil diameter. To describe the LC neuronal population activity, we utilized the coupled Lorenz models *X*_*i*_, *Y*_*i*_, *Z*_*i*_ (*i* = 1, 2) as follows:

(7) $$\begin{array}{r} \frac{dX_{i}}{dt}=a_{i}\left(Y_{i}-X_{i}\right)+J\left(X_{j}-X_{i}\right), \end{array}$$

(8) $$\begin{array}{r} \frac{dY_{i}}{dt}=c_{i}X_{i}-X_{i}Z_{i}-Y_{i}, \end{array}$$

(9) $$\begin{array}{r} \frac{dZ_{i}}{dt}=X_{i}Y_{i}-b_{i}Z_{i}, \end{array}$$

where, *X*_*i*_, *Y*_*i*_, *Z*_*i*_ represent the variable of Lorenz models coupled by strength of *J*. The index *i* distinguishes the left and right sides. We also assumed that the neural activities in the left and right LC weakly synchronize with each other. Therefore, we used the parameter set (*a*_*i*_ = 10, *b*_*i*_ = 8/3, *c*_*i*_ = 28, *J* = 0.7) to produce weakly synchronized state (correlation coefficient between *X*_1,2_: *R* ≈ 0.165). The initial values (*X*_1_, *Y*_1_, *Z*_1_) = (0.0, 1.0, 1.0) and (*X*_2_, *Y*_2_, *Z*_2_) = (0.0, 1.1, 1.1) were used. The LC neural population activities *x*_*i*_ (*i* = 1, 2) are then given by

(10) $$\begin{array}{r} x_{i}=L\;\text{zscore}\left(X_{i}\right)+b_{i}, \end{array}$$

where *L* and *b*_*i*_ are parameters. *b*_*i*_ is the LC baseline firing rate. In this study, *b*_1_ = *b*_2_ = *b*. zscore indicates the application of a z-score transformation. In the parasympathetic pathway, the outputs *S*_*i*_ from the EWN to the ciliary ganglion are as follows:

(11) $$\begin{array}{r} S_{i}=f\left(-w_{ii}x_{i}-w_{ji}x_{j}+\beta_{i}\right), \end{array}$$

where *w*_*ji*_ is the weight of the inhibitory synaptic connection from the LC *j* to EWN *i* and β_*i*_ represents all other inputs to EWN *i*. *f*(*x*) = tanh(*x*) + 1 is the activation function. The contralateral synaptic weights were set to *w*_12_ = *w*_21_ = *w*_*c*_. In the sympathetic pathway, the inputs to the superior cervical ganglion are given by

(12) $$\begin{array}{r} D_{i}=s_{i}x_{i}, \end{array}$$

where *s*_*i*_ denotes the synaptic weight of the LC connection to the superior cervical ganglion. Since, *D*_*i*_ and *S*_*i*_ correspond to dilator and sphincter muscle activation, respectively, the pupil diameter is proportional to

(13) $$\begin{array}{r} P_{i}=D_{i}-S_{i}+P_{0}, \end{array}$$

where *P*_0_ is the base diameter of the pupil. The source code for simulating the *P*_*i*_ can be found at the following address: https://github.com/SouNobukawa/neural_model_for_controlling_pupil_diameter. In this study, we set *w*_11_ = *w*_22_ = 0.3, *w*_12_ = *w*_21_ = *w*_*c*_, *s*_*i*_ = 0.3, β_1, 2_ = β, *P*_0_ = 3.0, and *L* = 1.5. Figure 4 shows the typical time-series of variables of this model in the case with β = 2.0, *b* = 4.8. In comparison with experimentally measured pupil diameters shown in Figure 1, although detailed behaviors in time-series of *P*_*i*_ were different, and complex and symmetric behaviors were similar. In the analysis for complexity and symmetricity, the evaluation duration was set to 10 ≤ *t* ≤ 300 to involve dynamical intermittent behaviors such as the transition between symmetric and asymmetric behaviors.

**Figure 4:**
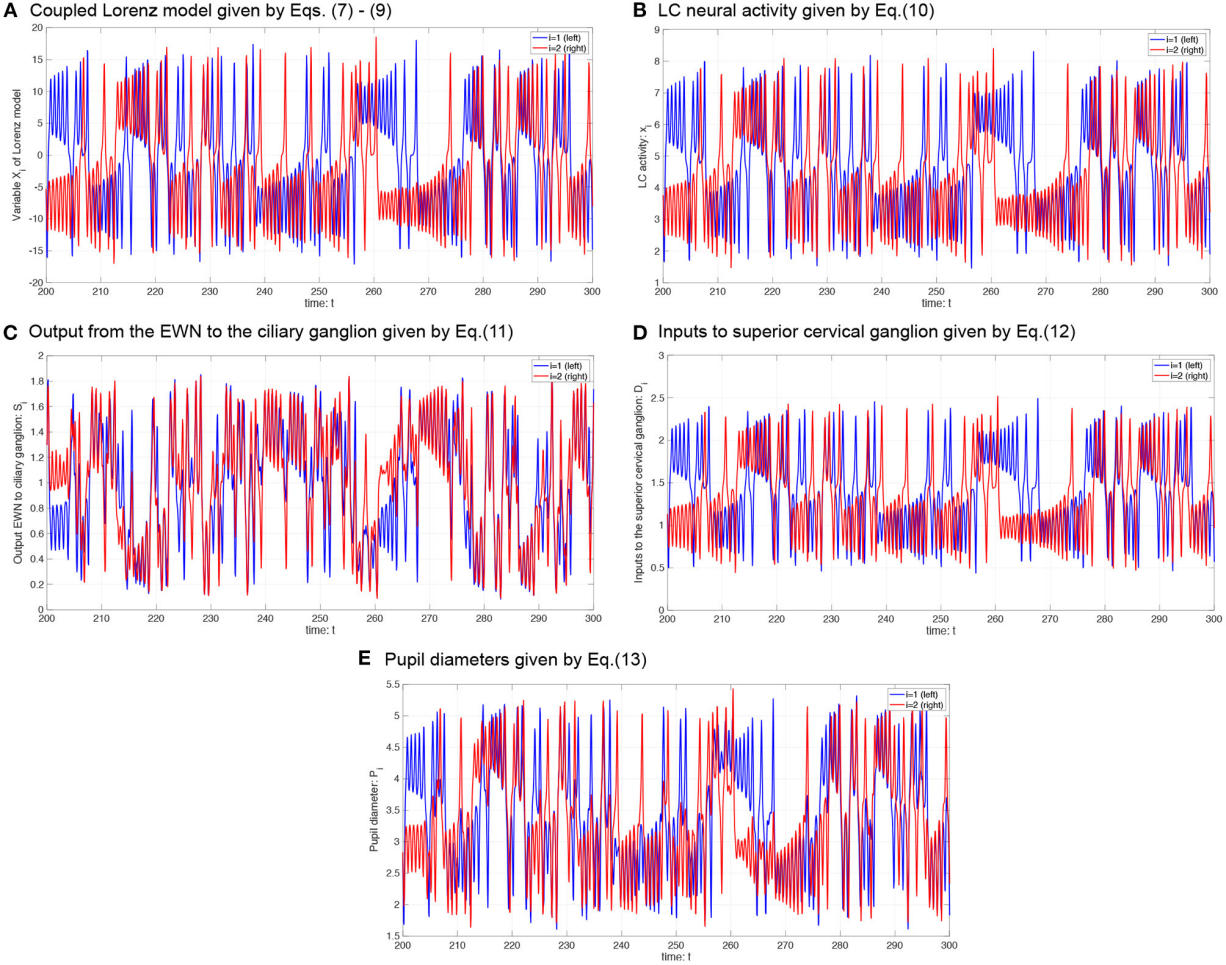
Typical time-series of neural model of the controller of pupil diameter. **(A)** Variable *X*_*i*_ in coupled Lorenz model. **(B)** Locus coeruleus (LC) activity *x*_*i*_. **(C)** Output from the Edinger-Westphal nucleus (EWN) to the ciliary ganglion *S*_*i*_. **(D)** Inputs to superior cervical ganglion *D*_*i*_. **(E)** Pupil diameters *P*_*i*_.

### 2.6. Surrogate Data Analysis

To investigate whether a non-linear dynamical process was involved in creating the observed pupil-diameter behavior, we generated surrogate data from a null hypothesis of no such involvement using the iterative amplitude-adjusted Fourier transformed (IAAFT) surrogate-data algorithm (Schreiber and Schmitz, 1996). The iteration number of the IAAFT procedure was 50. Ten IAAFT surrogate datasets were generated with different random seeds drawn from the pupil diameter measurements. The values of SampEn and TranEn calculated from these datasets were averaged and compared with the values derived from measurements.

### 2.7. Statistical Analysis

To compare the SampEn/TranEn of the measured time-series pupil data with those of the IAAFT surrogate data, we used a paired *t*-test. A two-tailed α level of 0.05 was adopted for statistical significance. If there was significant difference, then the time-series of the pupil-diameter was produced by a dynamical process and SampEn/TranEn could detect these dynamical properties.

To reveal relationships between the mean pupil diameter over time and SampEn or TranEn, a Gaussian-process regression (GPR), was used because the gaussian process is relatively robust for the data with inhomogeneous distribution (Williams and Rasmussen, 2006). Concretely, regression curves were estimated using a GPR trained on the all-epochs, all participants' datasets of temporal mean pupil diameter, and the corresponding SampEn or TranEn values. Moreover, the process for moving average was applied to the estimated fitting curves to clarify the profiles of SampEn/TranEn.

## 3. Results

### 3.1. Complexity and Symmetricity of Changes in Pupil Diameter

**Figure 5:**
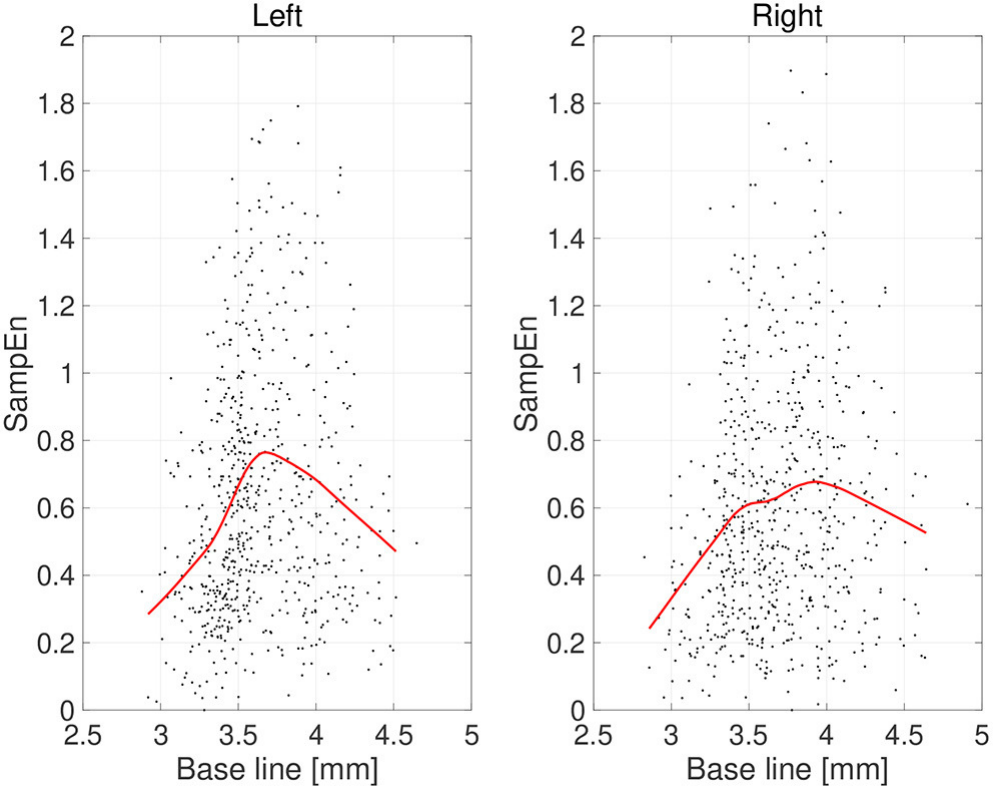
Scatter plots of sample entropy (SampEn) vs. the mean values of pupil diameter (baseline) calculated for 2-s epochs. Red line is a curve-fit conducted by a Gaussian process and moving average. An inverted-U dependence of SampEn on baseline is confirmed.

First, the temporal complexity of pupil diameter was evaluated. Figure 5 shows a scatterplot of the SampEn of all participants vs. the mean pupil diameters across time (baseline) together with regression curves estimated by GPR. When plotted against the baseline pupil diameter, which reflects the baseline activity of the LC (Rajkowski, 1993; Gilzenrat et al., 2003; Joshi et al., 2016; review in Aston-Jones and Cohen, 2005), SampEn exhibits an inverted-U shape. This relationship between complexity and baseline LC activity is congruent with previous findings (Usui and Stark, 1982). Moreover, an IAAFT surrogate data analysis against SampEn was conducted. Table 1 summarizes the results of comparing the mean SampEn values across epochs of pupil measurement with their IAAFT surrogate counterparts. The results confirm that the IAAFT procedure produces a significant enhancement of complexity, i.e., that the SampEn of pupil diameter reflects the presence of a deterministic process.

**Table 1:** Surrogate-data analysis of the complexity of pupil-diameter time series.

	**Original**	**Surrogate data**	**t-value (p-value)**
Left	0.63 (0.37)	0.94 (0.38)	**30.91(0.00)**
Right	0.60 (0.37)	0.92 (0.37)	**30.86(0.00)**

*Shown are the sample entropy (SampEn) of the original time series and of the corresponding iterative amplitude-adjusted Fourier transformed (IAAFT) surrogate time-series. Mean (standard deviation) values of SampEn across epochs are presented. Paired t-tests show significant enhancement of SampEn by the surrogate process. For clarity, values with p < 0.05 are shown in bold*.

**Figure 6:**
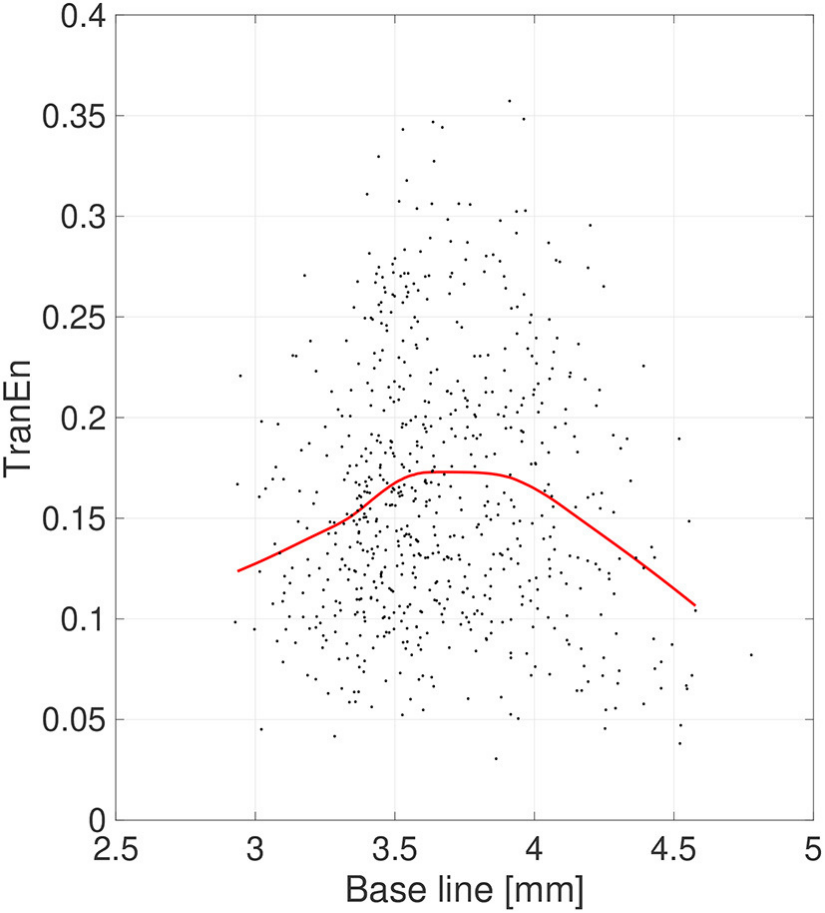
Scatter plots of transfer entropy (TranEn) vs. the mean values of pupil diameter (baseline) calculated for 2-s epochs. Here, the baseline values are averaged between right and left pupils and the TranEn values are averaged between the right-to-left and left-to-right cases. Red line is a curve-fit conducted by a Gaussian process and moving average. An inverted-U dependence of TranEn on baseline is confirmed.

Second, the symmetricity of the pupil diameters was investigated. Figure 6 shows the scatter plot of TranEn vs. the temporal mean value of pupil diameter (baseline) as well as the regression curve. Here, the baseline values were averaged between the right and left pupils, and the values of TranEn were averaged between the right-to-left and left-to-right cases. An inverted U-shape was also confirmed for the dependence of TranEn on baseline pupil diameter. Table 2 summarizes the values of TranEn averaged across epochs in the original time-series pupil data and in the corresponding IAAFT surrogate time-series. A significant enhancement of TranEn by the IAAFT process was confirmed. That is, like SampEn, TranEn reflects the contribution of a deterministic process.

**Table 2:** Surrogate-data analysis of the symmetricity of pupil-diameter time series.

**Original**	**Surrogate data**	**t-value (p-value)**
0.16 (0.06)	0.18 (0.05)	**11.4 (0.00)**

*Shown are the transfer entropy (TranEn) of the original time series and of the corresponding IAAFT surrogate time-series. Mean (standard deviation) values of TranEn across epochs are presented. Paired t-tests show significant enhancement of TranEn by the surrogate process. For clarity, values with p < 0.05 are shown in bold*.

### 3.2. Simulation of Pupil Behavior

**Figure 7:**
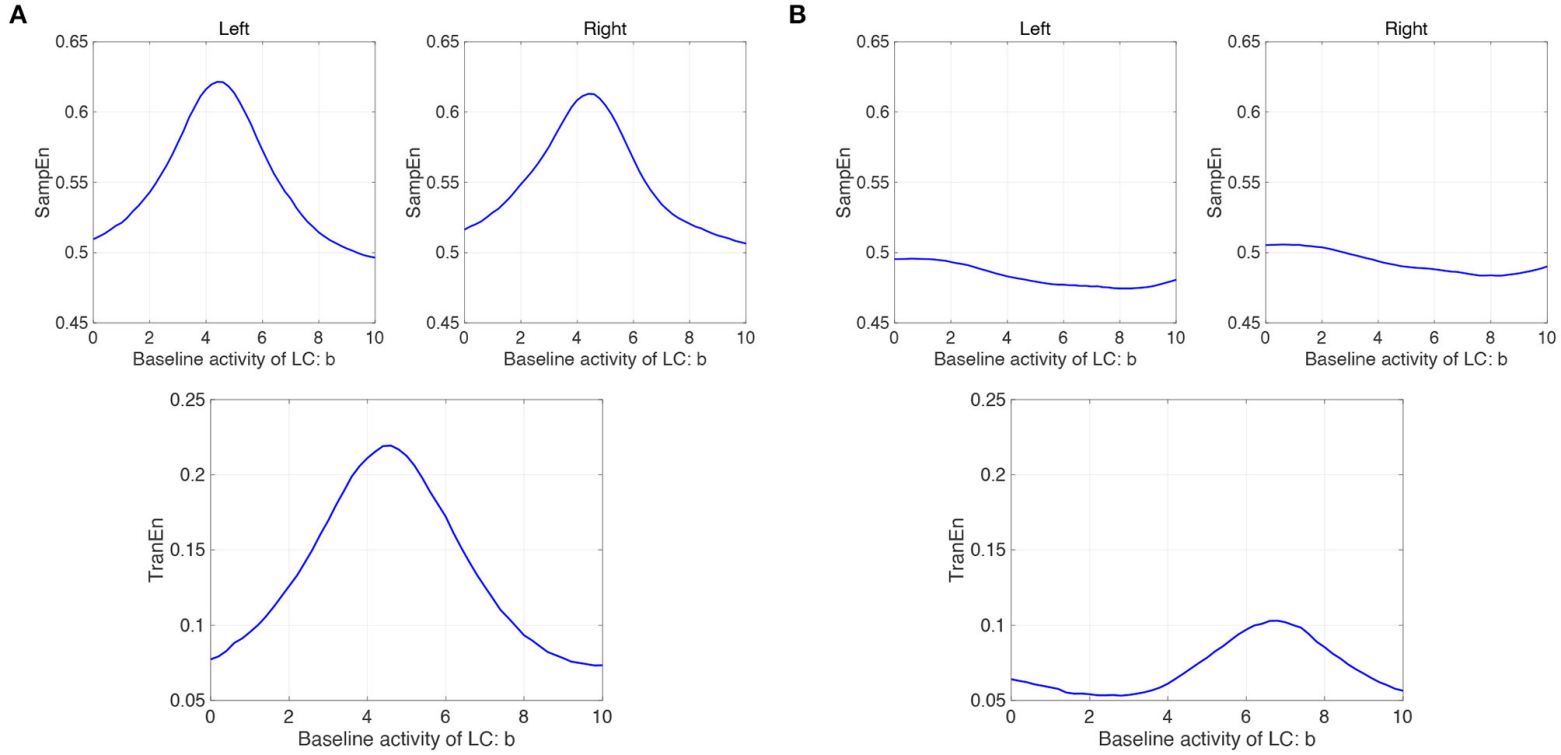
Modeling results: **(A)** (Upper parts) Dependence of SampEn in left and right eyes on the baseline activity of the locus coeruleus (LC) (denoted by *b*_1,2_ = *b* in Equation 10) in the case with the contralateral projection from the LC to the Edinger-Westphal nucleus (EWN) (denoted by *w*_12_ = *w*_21_ = *w*_*c*_ = 0.15 in Equation 11). (Lower part) Corresponding dependence of TranEn. SampEn and TranEn exhibit an inverted-U shape against baseline LC activity. **(B)** (Upper parts) Dependence of SampEn in left and right eyes on the baseline activity of the LC in the case without the contralateral projection from the LC to the EWN (*w*_12_ = *w*_21_ = *w*_*c*_ = 0 in Equation 11). (Lower part) Corresponding dependence of TranEn. SampEn does not exhibit definite peak; the peak of TranEn becomes smaller in comparison with the case with contralateral LC projections.

We evaluated the complexity and symmetricity of changes in pupil diameter calculated from a neural model of pupil control. Figure 7A shows the dependence of SampEn and TranEn on the synaptic weight on the baseline activity of the LC (denoted by *b*_1,2_ = *b* in Equation 10) in the case with the contralateral LC projections to the EWNs (denoted by *w*_12_ = *w*_21_ = *w*_*c*_ = 0.15 in Equation 11). Here, the other input to EWN instead of input from LC is fixed to β_1_ = β_2_ = β = 2.0. The results indicate that SampEn and TranEn exhibit an inverted U-shape against baseline LC activity. These peaks are SampEn≈ 0.62 and TranEn≈ 0, 22 at baseline LC activity *b* ≈ 4.8. While, the results in the case without the contralateral LC projections (*w*_*c*_ = 0) are shown in Figure 7B. SampEn does not exhibit a definite peak; the peak of TranEn becomes smaller in comparison with the case with contralateral LC projections of Figure 7A.

**Figure 8:**
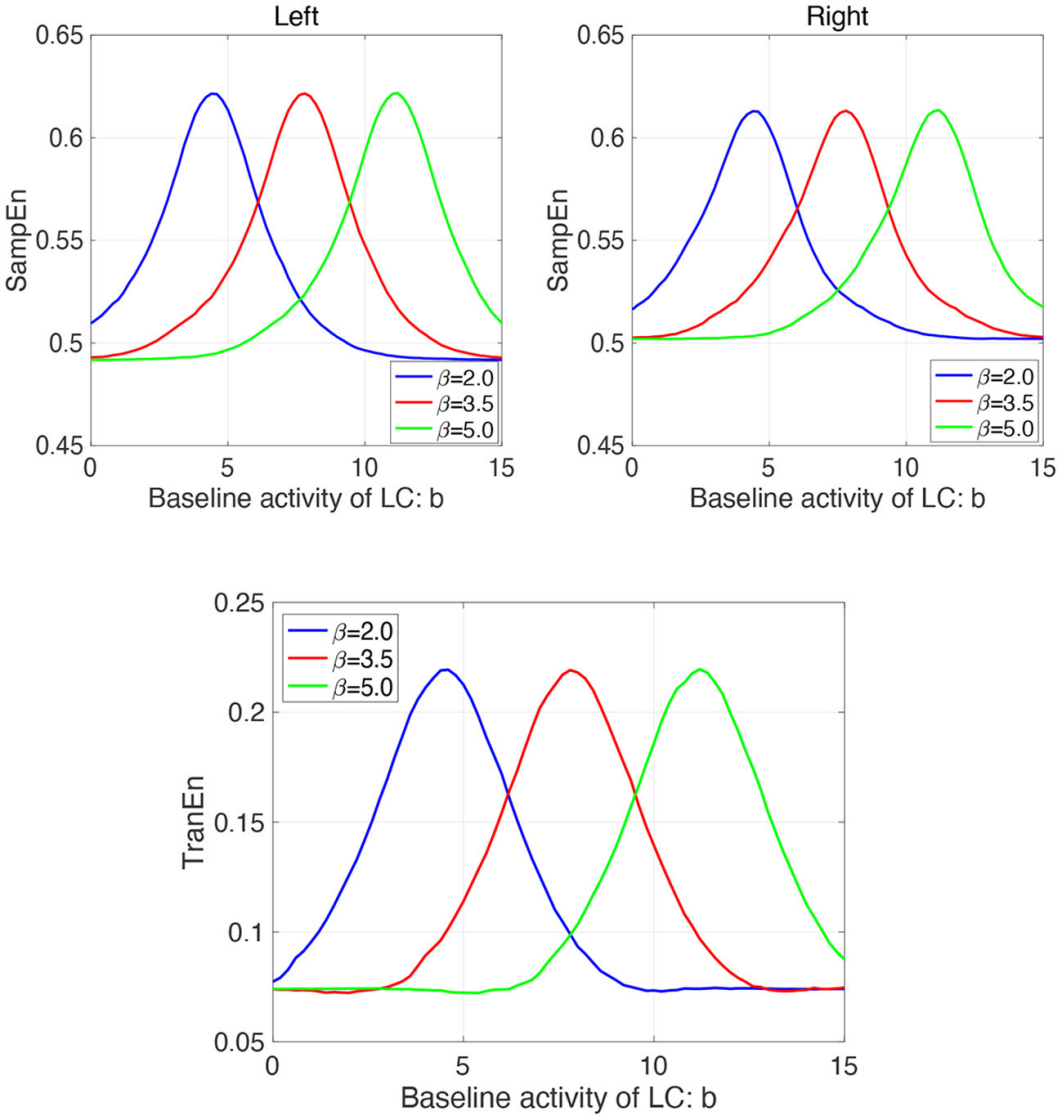
Modeling Results: (Upper parts) Dependence of SampEn in left and right eyes on the baseline activity of LC in the case with the other input to EWN instead of input from LC β_1_ = β_2_ = β = 2.0, 3.5, 5.0 under the contralateral projection from the LC to EWN *w*_12_ = *w*_21_ = *w*_*c*_ = 0.15. (Lower part) Corresponding dependence of TranEn. The peaks of SampEn and TranEn shift to the region of large *b*, increasing with β.

Furthermore, we investigate the influence of β on the profile of SampEn and TranEn under the contralateral LC projections to the EWNs. Figure 8 shows dependence of SampEn in left and right eyes on the baseline activity of LC in the case with β_1_ = β_2_ = β = 2.0, 3.5, 5.0 under the contralateral projection from the LC to EWN *w*_12_ = *w*_21_ = *w*_*c*_ = 0.15 and corresponding dependence of TranEn. Here, the β = 2.0 case corresponds to the result of Figure 7A. The peaks of SampEn and TranEn shift to the region of large *b*, increasing with β.

## 4. Discussion

In this study, we evaluated the complexity and symmetricity of the autonomous variations in pupil diameter in healthy participants. First, surrogate data analysis revealed that the complexity and symmetricity of changes in pupil diameter reflect the presence of a non-linear deterministic process. Because pupil diameters are controlled by parasympathetic and sympathetic pathways, this result implies that these complexity and symmetricity measures are physiologically inherent in the neural dynamics. Second, we investigated the relationships between the pupil-diameter baselines, which reflect tonic LC activity, and both the complexity and the symmetricity. The results indicated that both measures had a unimodal maximum at intermediate pupil diameters. Third, the simulation results from the neural model of pupil control showed that the non-linear threshold characteristics of the EWNs induce the observed inverted U-shaped profiles; this tendency is enhanced if the contralateral synaptic projections from the LC to the EWNs are above a certain strength.

The complexity and temporal patterns of pupil-diameter changes reflect LC activity, sleepiness, cognitive processes, autonomic nerve activity, and psychiatric disorders (Usui and Stark, 1982; Schumann et al., 2015; Artoni et al., 2019; Nakamura et al., 2019; Piu et al., 2019). Sleepiness in particular decreases the temporal complexity and determinicity of pupil diameter (Piu et al., 2019). Nakamura et al. reported that at the discrimination stage of the cognitive process, the complexity of pupil-diameter changes is reduced and pupil dilation occurs (Nakamura et al., 2019). Artoni et al. demonstrated that convolutional neural networks can detect disease-specific temporal patterns in the fluctuations of pupil diameter, a result obtained from mouse models of ASD. (Artoni et al., 2019). Moreover, studies on asymmetricity in pupil diameter have found that dilation appears strongly and independently in either pupil as a reaction to cognitive load (Kim et al., 1998; Kahya et al., 2018). Wahn et al. reported that the asymmetricity of pupil diameters also correlates with attentional load (Wahn et al., 2017). The above findings show that complexity and asymmetricity reflect a broad range of internal neural processes, thereby supporting our results on the emergence of specific complexity and symmetricity profiles against pupil baseline diameter, the latter correlating with LC activity.

The inverted U-shaped profiles of complexity and symmetricity against baseline activity require an explanation. This relationship was first reported by Usui and Stark (1982). Through simulations based on a neuronal model of the system controlling pupil diameter, these researchers found that the inverted U-shape is induced by a non-linear threshold characteristic of pupil dilation vs. the neural input signals to the system (Usui and Stark, 1982). In our model, this non-linear threshold characteristic corresponds to the EWN activation function *f*(*x*). More concretely, at low LC baseline activity, the output of the EWNs *S*_*i*_ converges to ≈ 2.0 and its variability is small. With increasing LC activity, *S*_*i*_ will be in the vicinity of the threshold of *f*(*x*) due to the inhibitory connections *w*_*ij*_ from the LC to the EWNs. In this state, the pupil diameters are driven by both dilator commands *D*_*i*_ and sphincter commands *S*_*i*_, resulting in large variability and peak complexity. Moreover, with appreciable synaptic weight at the contralateral connection to the EWNs, the contralateral LC activity *x*_*i*_ affects *S*_*i*_, enhancing the peak of complexity. With further increases in LC activity, *S*_*i*_ is ≈ 0.0, with the result that the pupil diameter is driven only by *D*_*i*_, resulting in decreased complexity. The inverted U-shape of symmetricity plotted against baseline LC activity is also induced by the EWN activation function. In the case of small LC baseline activity, common LC inputs to EWN through the contralateral synapses are weak, resulting in low symmetricity between the two EWNs *S*_1,2_ and thus between the two pupils. With increasing LC baseline activity, the LC drives the contralateral outputs to EWN *S*_1,2_ more strongly. The resulting common input to each side of the EWN enhances the symmetricity. With still greater LC activity, *S*_*i*_ is ≈ 0.0, and the pupils are driven only by the sympathetic pathway, which has no contralateral projections. Here, in larger other input to EWN instead of LC (represented by β_*i*_ in Equation 11), greater LC activity is needed to realize *S*_*i*_ ≈ 0.0. Therefore, the symmetricity decreases.

The advantages of evaluating complexity and symmetricity as SampEn and TranEn, respectively, requires further discussion. Conventional evaluation methods for pupil complexity and symmetricity utilize temporal standard deviation, Shannon entropy, and the difference between right and left pupil diameters (Usui and Stark, 1982; Poynter, 2017; Wahn et al., 2017; Piu et al., 2019). Moreover, Piu et al. showed that the combination of complexity estimation by Shannon entropy and determinism is an effective diagnostic tool for identifying the states of neural systems (Piu et al., 2019). The importance of determinism for evaluation is supported by the fact that changes in pupil diameter are produced by multiple non-linear neural pathways (Usui and Stark, 1982; Liu et al., 2017). However, the temporal standard deviation, Shannon entropy, and difference in pupil diameters cannot capture contributions from deterministic processes (Theiler et al., 1991; Schreiber and Schmitz, 1996). The SampEn and TranEn measures utilized in this study can precisely detect the contribution of determinism to pupil diameter changes, in addition to permitting evaluations of complexity and symmetricity (see Tables 1, 2). Therefore, it can be assumed that evaluation by SampEn and TranEn is a useful method for estimating the states of neural systems and deficits in neural function.

This study has several limitations. We revealed the typical profiles of complexity and symmetricity for autonomous pupillary changes. However, the nature of these change profiles in the presence of cognitive tasks and attentional loads remains unclear. Moreover, we only studied healthy participants in this study. It is imperative to measure the corresponding profiles in patients with psychiatric disorders involving deficits of attention and arousal, and those involving imbalances in exploration-exploitation, such as schizophrenia, ASD, and ADHD. In the model-based analysis, we modeled the LC activity as two neural populations sustaining weakly synchronized, chaotic oscillations. Recent progress in understanding the neural activity of the LC revealed that this activity exhibits complex spatio-temporal behaviors among multiple neural populations (Totah et al., 2019). However, the population size used in the present study is too small to describe these spatio-temporal behaviors. Thus, it will be necessary to increase the size of the neural populations or utilize cellular non-linear networks (Arena et al., 2005; Bucolo et al., 2008) and spiking neural networks (Nobukawa et al., 2019, 2020) with high physiological validity. We are currently planning to address these queries in future works.

## 5. Conclusions

In this study, we characterized the typical relationships between baseline activity, complexity, and symmetricity for resting-state changes in pupil diameter using the measures SampEn and TranEn by analyzing pupil diameters and constructing a model controller of pupil diameter. Our proposed evaluation method and our findings may find application in the development of pupillometric estimation and diagnostic tools for application to healthy brain states, their functions, and to psychiatric disorders.

## Data Availability Statement

The datasets presented in this article are not readily available because the datasets generated for this study will not be made publicly available because the informed consent did not include the declaration regarding publicity of clinical data. Requests to access the datasets should be directed to Sou Nobukawa, nobukawa@cs.it-chiba.ac.jp.

## Ethics Statement

The studies involving human participants were reviewed and approved by Ethics Committee of Showa University. The patients/participants provided their written informed consent to participate in this study.

## Author Contributions

SN, AS, TTaka, and ST conceived the methods. SN, AS, TTaka, MK, and ST analyzed and discussed the results. SN, AS, TTaka, and ST wrote the main manuscript and prepared all figures. AS, TTake, HO, AI, NK, and ST conducted the experiments. All authors contributed to manuscript revision and have read and approved the submitted version.

## Conflict of Interest

The authors declare that the research was conducted in the absence of any commercial or financial relationships that could be construed as a potential conflict of interest.
